# Urinary Tract Infection, Bacteremia, and Meningitis Among Febrile Young Infants With SARS-CoV-2 and Non–SARS-CoV-2 Viral Infections

**DOI:** 10.1001/jamanetworkopen.2023.21459

**Published:** 2023-06-29

**Authors:** Brett Burstein, Alexandra Yannopoulos, Kelley-Anne Dionne

**Affiliations:** 1 Division of Pediatric Emergency Medicine, Montreal Children’s Hospital, McGill University Health Centre, Montreal, Quebec, Canada; 2 Department of Biostatistics, Epidemiology and Occupational Health, McGill University, Montreal, Quebec, Canada; 3 Research Institute of the McGill University Health Centre, Montreal, Quebec, Canada

## Abstract

This cohort study assesses the prevalence of urinary tract infections (UTIs), bacteremia, and bacterial meningitis among infants systematically tested for SARS-CoV-2 and non–SARS-CoV-2 viruses, with a focus on invasive bacterial infections.

## Introduction

Guidelines from the American Academy of Pediatrics (AAP) have been published to evaluate febrile infants aged 8 to 60 days for urinary tract infections (UTIs), bacteremia, and bacterial meningitis.^1^ Studies^2^ suggest that the presence of a respiratory virus is associated with decreased risk of these infections; however, it remains unclear how viral testing should guide laboratory evaluation and management.^1^ Moreover, the emergence of SARS-CoV-2 and changing epidemiology since the COVID-19 pandemic present further challenges to risk stratification.^3^ This study sought to assess the prevalence of UTIs, bacteremia, and bacterial meningitis among infants systematically tested for SARS-CoV-2 and non–SARS-CoV-2 viruses, with a focus on invasive bacterial infections (IBIs; ie, bacteremia and bacterial meningitis collectively).^1^

## Methods

This was a secondary analysis of prospectively collected quality improvement data for all infants aged 60 days and younger evaluated for fever at an urban tertiary pediatric emergency department between March 2020 and December 2022.^4^ Standardized clinical, laboratory, and telephone follow-up data were collected for all infants. Only previously healthy, full-term infants aged 8 to 60 days with a documented rectal temperature of 38.0 °C or higher meeting AAP inclusion and exclusion criteria were included for analysis.^1^ All infants underwent multiplex respiratory testing that included SARS-CoV-2 (eTable in Supplement 1). Cerebrospinal fluid testing was at the discretion of the treating physician. Final infection status was confirmed for all infants by microbiologic culture results and telephone follow-up 14 to 28 days after discharge. UTI, bacteremia, and bacterial meningitis were defined according to current definitions.^2,3^ Infants with SARS-CoV-2 plus any other non–SARS-CoV-2 virus detected were analyzed in the SARS-CoV-2 group.

Prevalences of any infection (UTI, bacteremia, and bacterial meningitis) and IBI specifically were compared between infants with no virus detected and SARS-CoV-2 or non–SARS-CoV-2 infections by χ^2^ testing using Stata statistical software version 14.1 (StataCorp). A 2-tailed *P *< .05 was considered statistically significant.

This cohort study received approval from the McGill University Heath Centre research ethics board, with a waiver of informed consent because the data were collected prospectively for quality improvement purposes. The study followed Strengthening the Reporting of Observational Studies in Epidemiology (STROBE) reporting guideline.

## Results

There were 931 infants included for analysis. Their median (IQR) age was 38 (25-49) days, 547 (58.8%) were male, and 428 (46.0%) were hospitalized (Table). Almost all infants had culture results available for blood (871 infants [93.6%]) and urine (875 infants [94.0%]). Overall, 107 infants (11.5%) had UTI, bacteremia, or bacterial meningitis, and 20 (2.2%) had IBIs. Viruses were detected among 611 infants (65.6%), including 163 (17.5%) with SARS-CoV-2. The prevalence of UTI, bacteremia, and bacterial meningitis was lower among infants with non–SARS-CoV-2 viruses (35 of 448 infants [7.8%]) compared with those with no detectable virus (67 of 320 infants [20.9%]); however, these infections were least prevalent among infants with SARS-CoV-2 (5 of 163 infants [3.1%]) (Figure). All 5 infections concomitant with SARS-CoV-2 were culture-confirmed UTIs among infants aged 41 to 59 days with positive urinalyses at initial evaluation. There were significantly fewer IBIs in both SARS-CoV-2 (0 of 163 infants) and non–SARS-CoV-2 groups (5 of 448 infants [1.11%]) compared with virus-negative infants (15 of 320 infants [4.69%]). All 5 IBIs concomitant with non–SARS-CoV-2 viruses were bacteremia among infants aged 11 to 56 days.

**Table.** Clinical and Laboratory Characteristics of Infants by Viral Infection Status

Characteristic	Infants, No. (%)			
	Total (N = 931)	No viral infection (n = 320)	Non–SARS-CoV-2 viral infection (n = 448)^a^	SARS-CoV-2 infection (n = 163)^b^
Sex				
Male	547 (58.8)	194 (60.6)	254 (56.7)	99 (60.7)
Female	384 (41.2)	126 (39.4)	194 (43.3)	64 (39.3)
Age, d				
8-21	164 (17.6)	85 (26.6)	58 (12.9)	21 (12.9)
22-28	124 (13.3)	54 (16.9)	55 (12.3)	15 (9.2)
29-60	643 (69.1)	181 (56.6)	335 (74.8)	127 (77.9)
Hospitalized	428 (46.0)	186 (58.1)	199 (44.4)	43 (26.4)
Cerebrospinal fluid culture available	329 (35.3)	165 (55.3)	135 (30.1)	29 (17.8)
Maximal temperature, median (IQR), °C				
All infants	38.3 (38.1-38.7)	38.4 (38.1-38.9)	38.3 (38.1-38.6)	38.4 (38.1-38.7)
Infants with UTI, bacteremia, or bacterial meningitis	38.8 (38.3-39.2)	38.9 (38.5-39.3)	38.5 (38.2-39.0)	38.3 (38.2-39.0)
Procalcitonin, median (IQR), μg/L^c^				
All infants	0.1 (0.1-0.2)	0.1 (0.1-0.4)	0.1 (0.1-0.2)	0.1 (0.1-0.2)
Infants with UTI, bacteremia, or bacterial meningitis	0.7 (0.2-5.1)	1.6 (0.4-7.8)	0.3 (0.1-0.8)	0.1 (0.1-0.2)
C-reactive protein, median (IQR), mg/dL^d^				
All infants	0.31 (0.09-1.12)	0.29 (0.05-2.28)	0.48 (0.16-1.17)	0.14 (0.07-0.28)
Infants with UTI, bacteremia, or meningitis	2.90 (0.94-6.50)	3.94 (1.32-6.91)	2.01 (0.93-6.18)	0.17 (0.14-0.23)
Absolute neutrophil count, median (IQR), cells/μL^e^				
All infants	2700 (1800-4500)	2800 (1800-5300)	3100 (2000-4600)	1900 (1300-2700)
Infants with UTI, bacteremia, or bacterial meningitis	5600 (2900-8400)	6600 (3400-9500)	4600 (2700-6500)	1800 (1600-2200)
Bacterial infections	107 (11.5)	67 (20.9)	35 (7.8)	5 (3.1)
UTI	87 (9.3)	52 (16.3)	30 (6.7)	5 (3.1)
Bacteremia	5 (0.54)	3 (0.94)	2 (0.45)	0
Meningitis	1 (0.11)	1 (0.31)	0	0
UTI plus bacteremia	12 (1.3)	9 (2.8)	3 (0.67)	0
UTI plus meningitis	1 (0.11)	1 (0.31)	0	0
Bacteremia plus meningitis	1 (0.11)	1 (0.31)	0	0

Abbreviation: UTI, urinary tract infection.

SI conversion factors: To convert C-reactive protein to milligrams per liter, multiply by 10; neutrophils to cells ×10^9^ per liter, multiply by 0.001.

^a^ A total of 67 infants (15.0%) had 2 non–SARS-CoV-2 viral infections; 3 infants (0.7%) had 3 or more non–SARS-CoV-2 viral infections.

^b^ A total of 22 infants (13.5%) had SARS-CoV-2 plus 1 viral coinfection (11 had SARS-CoV-2 plus human rhinovirus or enterovirus, 3 had SARS-CoV-2 plus respiratory syncytial virus, 2 had SARS-CoV-2 plus human metapneumovirus, 1 had SARS-CoV-2 plus coronavirus 229E, 2 had SARS-CoV-2 plus adenovirus, 2 had SARS-CoV-2 plus parainfluenza virus 4, and 1 had SARS-CoV-2 plus parainfluenza virus 3); 1 infant (0.7%) had SARS-CoV-2 plus 2 or more viral coinfections (1 had SARS-CoV-2 plus adenovirus plus human rhinovirus or enterovirus).

^c^ Procalcitonin was available for 843 of 931 infants (90.5%).

^d^ C-reactive protein was available for 829 of 931 infants (89.0%).

^e^ Absolute neutrophil count was available for 867 of 931 infants (93.1%).

**Figure.**
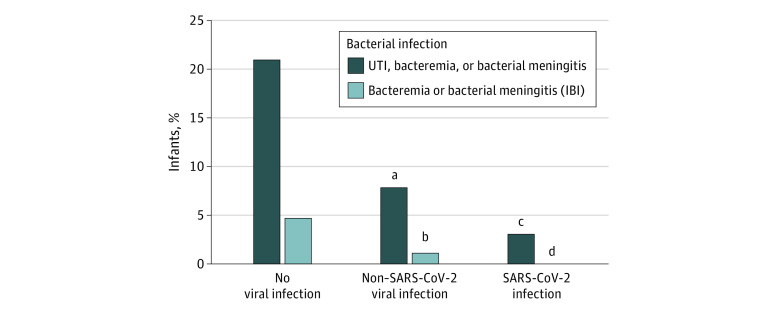
Prevalence of Any Infection and Invasive Bacterial Infections (IBIs) Specifically Among Febrile Infants According to Viral Status UTI indicates urinary tract infection. ^a^*P* < .001 vs no viral infection group. ^b^*P* = .002 vs no viral infection group. ^c^*P* = .04 vs non–SARS-CoV-2 viral infection group. ^d^*P* = .005 vs no viral infection group.

## Discussion

To our knowledge, this cohort study is the first to assess the prevalence of UTI, bacteremia, and bacterial meningitis among febrile young infants systematically tested for viral infections including SARS-CoV-2. Findings are similar to previous studies with selective viral testing, suggesting a significantly lower but nonnegligible risk of UTI and IBI among infants with non–SARS-CoV-2 infections^1,2^ and very low risk of IBI among those with SARS-CoV-2.^5^ These findings support AAP recommendations that a confirmed non–SARS-CoV-2 virus should not affect the initial evaluation for young infants with fever.^1^ However, findings demonstrating the very low risk of IBIs among infants with SARS-CoV-2 may assist clinicians individualize management and inform shared decision-making with parents, particularly when rapid COVID-19 testing results are known or available at the point of care.

A limitation of this study is that it was conducted at a single center. SARS-CoV-2 variant testing was uncommon, and variants may not be equally associated with lower infection risk. Multiplex testing does not distinguish between human rhinovirus and enterovirus, which could underestimate the difference between virus-negative and non–SARS-CoV-2 groups.^6^ Only 20 infants had IBIs, but the study cohort was similarly large compared with prospective cohorts informing AAP guidelines.^1^

## Notes

Supplement 1. **eTable.** BioFire Respiratory Panel 2.1 (RP2.1)

Supplement 2.
Data Sharing Statement
