# Supplementary material for: Urinary Tract Infection, Bacteremia, and Meningitis Among Febrile Young Infants With SARS-CoV-2 and Non–SARS-CoV-2 Viral Infections
Source: JAMA Netw Open. 2023 Jun 29;6(6):e2321459. doi: 10.1001/jamanetworkopen.2023.21459 (PMC10311385; doi:10.1001/jamanetworkopen.2023.21459)
Supplement: Supplement 1. — eTable. BioFire Respiratory Panel 2.1 (RP2.1) [file jamanetwopen-e2321459-s001.pdf]

Supplemental Online Content

Burstein B, Yannopoulos A, Dionne KA. Urinary tract infection, bacteremia, and meningitis among febrile young infants with SARS-CoV-2 and non–SARS-CoV-2 viral infections. *JAMA Netw Open*. 2023;6(6):e2321459. doi:10.1001/jamanetworkopen.2023.21459

eTable. BioFire Respiratory Panel 2.1 (RP2.1)

This supplemental material has been provided by the authors to give readers additional information about their work.

© 2023 Burstein B et al. *JAMA Network Open.*

eTable: BioFire Respiratory Panel 2.1 (RP2.1)
**Viruses**
Adenovirus
Coronavirus 229E
Coronavirus HKU1
Coronavirus NL63
Coronavirus OC43
SARS-CoV-2
Human Metapneumovirus
Human Rhinovirus/Enterovirus
Influenza A
Influenza B
Parainfluenza Virus 1
Parainfluenza Virus 2
Parainfluenza Virus 3
Parainfluenza Virus 4
Respiratory Syncytial Virus
**Bacteria**
Bordetella parapertussis
Bordetella pertussis
Chlamydia pneumoniae
Mycoplasma pneumoniae

© 2023 Burstein B et al. *JAMA Network Open.*
