# Supplementary material for: Urinary Tract Infection, Bacteremia, and Meningitis Among Febrile Young Infants With SARS-CoV-2 and Non–SARS-CoV-2 Viral Infections
Source: JAMA Netw Open. 2023 Jun 29;6(6):e2321459. doi: 10.1001/jamanetworkopen.2023.21459 (PMC10311385; doi:10.1001/jamanetworkopen.2023.21459)
Supplement: Supplement 2. — Data Sharing Statement [file jamanetwopen-e2321459-s002.pdf]

Data Sharing Statement

Burstein. Urinary Tract Infection, Bacteremia, and Meningitis Among Febrile Young Infants With SARS-CoV-2 and Non–SARS-CoV-2 Viral Infections. *JAMA Netw Open*. Published June 29, 2023. doi:10.1001/jamanetworkopen.2023.21459

Data
Data available: No

Additional Information
Explanation for why data not available: Not approved by institutional REB for public data sharing of individual-level data
